# Effects of acute static stretching and dynamic warm-up protocols on shoulder function in young adult male athletes with shoulder impingement syndrome: a randomized controlled crossover trial

**DOI:** 10.1186/s12891-025-09379-0

**Published:** 2025-12-20

**Authors:** Mohammad Alghosi, Maryam Abduljabbar Khudhair, Mohammed Sadique K, Fereshteh Ejlali, Mojtaba Iranmanesh, Mohammad Alimoradi, Nicola Relph, Ali Shamsi Majelan

**Affiliations:** 1 Department of Physical Education, Technical and Vocational University (TVU), Tehran, Iran; 2 College of Physical Education and Sports Sciences for Girls, University of Baghdad, Baghdad, Iraq; 3 Department of Physical Education and Sports, Pondicherry University, Puducherry, India; 4 Department of Physical Education, Muhammed Abdurahiman Memorial Orphanage College, Mukkam, Kerala India; 5 Department of Physical Education and Exercise Science, Central Tehran Branch (Velayat Campus), Islamic Azad University, Tehran, Iran; 6 Department of Sports Injuries and Corrective Exercises, Faculty of Sports Sciences, Shahid Bahonar University of Kerman, Kerman, Iran; 7 Exercise & Research Center, HERC – Health, Mina Rashid, Dubai Maritime City, Dubai, United Arab Emirates; 8 Faculty of Health, Social Work and Medicine, Edge Hill University, Ormskirk, Lancashire, UK; 9 Department of Sports Injury and Corrective Exercise, Faculty of Physical Education and Sport Sciences, University of Guilan, Rasht, Iran

**Keywords:** Shoulder impingement syndrome, Athlete, Stretching, Dynamic warm-up, Performance

## Abstract

**Background:**

Shoulder impingement syndrome (SIS) is common among overhead athletes. Static stretching (SS) and dynamic warm-up (DW) are widely used, but their acute effects on comprehensive shoulder function in athletes with SIS are not fully understood. This study compared the acute effects of SS, DW, and a combined protocol (SS + DW) on range of motion (ROM), stability, proprioception, and strength in male athletes with SIS and healthy controls.

**Methods:**

In this randomized controlled crossover trial, 25 male athletes with SIS and 25 healthy controls performed SS, DW, and SS + DW protocols in a randomized order. Outcomes included shoulder internal rotation (IR) and external rotation (ER) ROM, Y-Balance Test (YBT) performance, joint position sense (JPS) accuracy, and isokinetic strength, measured at baseline, immediately post-intervention, and an hour follow-up. Data were analyzed using mixed-design ANOVA.

**Results:**

All protocols significantly improved IR and ER ROM (*p* < 0.05), with SS producing the greatest gains for the SIS group (IR + 4.4°, *d* = 1.25; ER + 3.4°, *d* = 1.02). For the SIS group, DW resulted in the largest improvements in YBT performance (+ 7.4 cm, *d* = 1.09; *p* < 0.05) and markedly enhanced JPS accuracy (error reduction of − 3.8°, *d* = 6.66; *p* < 0.05). In contrast, SS increased proprioceptive error in SIS athletes (+ 1.2°, *d* = 2.91; *p* < 0.05). Isokinetic strength analysis showed that SS significantly reduced eccentric IR strength at 60°/s (–2.5 Nm, *d* = 0.69; *p* < 0.05), whereas DW improved concentric ER strength at 60°/s (+ 0.6 Nm, *d* = 0.13; *p* < 0.05). Across all outcomes, the combined protocol produced effect sizes ranging from *d* = 0.08 to 3.06, representing small to large effects (*p* < 0.05).

**Conclusions:**

The findings indicate that while all warm-ups improve ROM and stability, DW offers the most comprehensive benefits for athletes with SIS by enhancing proprioception and strength without the performance inhibition associated with SS. DW is recommended for pre-activity routines when neuromuscular readiness is critical.

**Trial registration:**

This trial was registered prospectively at the Iranian Registry of Clinical Trials (Identifier: IRCT20230612058457N6) on June 4, 2025.

**Supplementary Information:**

The online version contains supplementary material available at 10.1186/s12891-025-09379-0.

## Background

The shoulder joint is one of the most mobile and complex joints in the human body, enabling athletes to perform a broad spectrum of sports skills [1, 2]. This mobility, however, predisposes the shoulder to a high risk of injury, particularly in athletes involved in overhead sports that demand repetitive, forceful arm movements [2]. Preventive and rehabilitative interventions are vital for maintaining shoulder health, reducing the incidence of injuries, and enhancing athletic performance [3]. Stretching and warm-up protocols are widely recommended components in both sports training and rehabilitation strategies [4]. Acute static stretching (SS) involves holding muscles in an elongated position to promote flexibility and relaxation, whereas dynamic warm-up (DW) consists of sport-specific active movements aimed at increasing blood flow, improving flexibility, and stimulating neuromuscular activation [5, 6]. Both approaches, however, may have different effects on shoulder biomechanics and function in individuals with SIS, highlighting the need for direct comparison, combination, and application-specific guidance [7].

More recently, DW may be preferred by athletes and trainers based on evidence demonstrating its positive or neutral effect on muscular performance compared to the sometimes detrimental effects of prolonged SS on strength and speed, particularly before tasks requiring power output [7]. Systematic reviews support DW as an optimal pre-exercise strategy to enhance muscular readiness and potentially reduce injury risk, although the evidence on injury prevention remains mixed [8]. Furthermore, direct comparisons of SS and DW protocols, such as those incorporating elastic bands, have shown both can acutely improve shoulder range of motion (ROM), with SS improving internal rotation (IR) gains slightly more than DW. Therefore, combined approaches may help mitigate the negative influence of isolated SS on motor performance, as demonstrated in female handball and other overhead athletes [9], although research on combination protocols in athletes with pre-existing injuries is lacking.

Despite advances in understanding shoulder pathomechanics, one such shoulder injury, shoulder impingement syndrome (SIS), remains a prevalent and challenging condition to manage, frequently associated with modifiable factors such as glenohumeral internal rotation deficit (GIRD), altered scapulohumeral kinematics, and pain-induced neuromuscular inhibition [10, 11]. Among overhead athletes, the incidence rate of SIS ranges from 0.2 to 1.8 cases per 1,000 h of practice, with a prevalence of 5% to 36% [12]. Effective interventions targeting shoulder function are crucial, given the negative impact of SIS on athletes’ ROM, strength, and overall performance [11, 13]. In athletes presenting with GIRD, rehabilitation programs integrating stretching, mobility enhancement, and strengthening exercises are effective in improving ROM and shoulder function [14–16]. These programs may also increase the subacromial space, a key factor implicated in the development of SIS. Meta-analytic evidence further associates GIRD with elevated injury risk, emphasizing the importance of early intervention to restore shoulder movement balance prior to exposure to increased training and competition demands [14].

Despite these insights, evidence on the acute effects of SS and DW on shoulder stability and proprioception, especially joint position sense (JPS), remains limited. Most research has focused on ROM and functional performance, with less attention to neuromuscular control measures critical for injury prevention and rehabilitation. Assessments such for determining whether DW’s neuromuscular benefits lead to meaningful improvements in stability for athletes with SIS [17].

Given the current gaps, this randomized controlled crossover trial (RCCT) aims to determine the immediate effects of SS and DW on shoulder function in male athletes with SIS, focusing on ROM, strength, stability, and JPS. Recent guidelines advocate exercise-based conservative management as a frontline strategy for SIS [18], while evidence suggests that DW alone or in combination with SS to maintain performance and enhance mobility in acute settings [19]. However, comparative data on neuromuscular outcomes remain scarce, particularly within the SIS athlete population. This study thus addresses an important knowledge gap, providing evidence to optimize rehabilitation and training interventions that support shoulder health and athletic performance.

It was expected that all warm-up protocols would acutely improve shoulder ROM and stability in athletes with SIS. However, we hypothesized that the DW would result in greater improvements in proprioception, balance, and isokinetic strength compared with SS, while the SS + DW would yield intermediate effects by enhancing flexibility without compromising neuromuscular performance.

## Methods

### Study design and setting

**Fig. 1:**
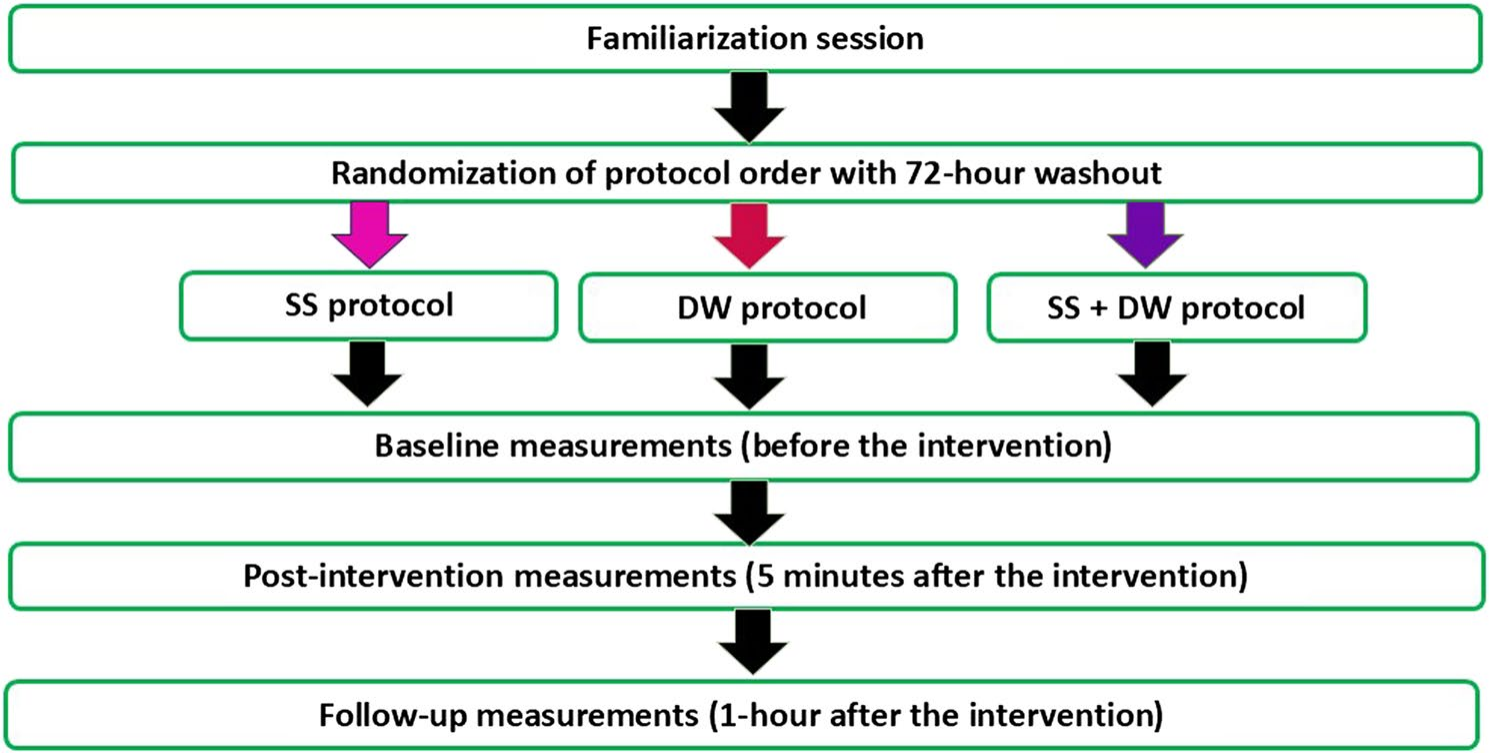
Schematic of the experimental protocol. Each participant completed three interventions in randomized order. Each session included baseline, post-intervention, and 1-hour follow-up assessments, separated by a 72-hour washout after a familiarization session. Abbreviations: SS, static stretching; DW, dynamic warm-up; SS + DW, combination of static stretching and dynamic warm-up

This RCCT was conducted at the Faculty of Education and Psychology, Shiraz University, Iran, from June 9 to June 18, 2025, to assess the acute effects of SS, DW, and their combination (SS + DW) on shoulder function in male athletes (aged 20–30) with SIS. Participants were recruited from volleyball, handball, and basketball teams. Using a computer-generated Latin square randomization design, participants completed the three intervention protocols in a counterbalanced order, ensuring that each protocol occurred equally in each sequence position to minimize potential order and carryover effects. Although participants were aware of the intervention being performed, the assessor responsible for data collection and analysis was blinded to the protocol order. Each protocol included three standardized assessment time points: baseline (pre-test), immediately after the intervention (post-intervention), and a 1-hour follow-up within the same session. All sessions were spaced 72 h apart to allow full recovery and minimize carryover effects, following an initial familiarization session (Fig. 1). The study was conducted in a controlled laboratory environment using standardized equipment. Although the crossover design enabled within-subject comparisons, a healthy control group was included to provide a normative reference. This allowed differentiation between intervention effects specific to SIS-related deficits and general responses observed in individuals with normal shoulder function. Primary outcomes included shoulder ROM, stability, proprioception, and isokinetic strength. The study was approved by the Research Ethics Committee of the University of Guilan, Iran (Code: IR.GUILAN.REC.1404.035) on May 19, 2025, and prospectively registered in the Iranian Registry of Clinical Trials (IRCT20230612058457N6) on June 4, 2025. All participants provided written informed consent after being informed of any potential risks associated with the procedures. The study was conducted in accordance with the principles of the Declaration of Helsinki. This study adheres to the CONSORT (Consolidated Standards of Reporting Trials) guidelines for the reporting of randomized crossover trials [20]. The completed checklist is provided in Supplementary File 1.

### Participants

Fifty young adult male athletes (age: 25.7 ± 1.5 years; height: 197.5 ± 4.1 cm; mass: 92.3 ± 3.5 kg) participated in this study. Twenty-five athletes diagnosed with SIS by a certified sports medicine physician, confirmed by at least two clinical signs (e.g., painful arc, Neer or Hawkins-Kennedy tests, resisted external rotation (ER) pain, or positive apprehension-relocation test without posterior pain) [21, 22], were recruited as the experimental group. Only participants with impingement in their dominant limb, as reported during screening, were included. Twenty-five healthy athletes with no history or signs of SIS were included as controls for comparative analysis. All participants were active members of sports teams in Shiraz, Iran. Eligibility criteria included being aged 20–30 years, physically active, and having not participated in any shoulder-specific training programs in the past month. Athletes were excluded if they had a history of shoulder surgery or dislocation, were currently undergoing physiotherapy or medication, or had performed strenuous exercise within 48 h prior to testing. The sample size was calculated a priori using G*Power software (version 3.1.9.7, Heinrich-Heine-Universität Düsseldorf, Germany). The calculation was based on a mixed-design analysis of variance (ANOVA) (within-between interaction). The effect size was set to f = 0.23, which is considered a small effect size according to conventional guidelines [23]. This value was derived from pilot data on shoulder ROM changes after SS and DW in athletes with SIS. This conservative estimate was chosen to ensure the study would be sufficiently powered to detect a clinically meaningful effect, even if it was modest [24]. Using an alpha level (α) of 0.05 and a desired power (1-β) of 0.90, the analysis indicated a minimum required sample size of 21 participants per group. To compensate for potential participant dropout, the sample size was increased by 20%, resulting in a final recruitment target of 25 participants per group [25].

### Intervention protocol

To examine the acute impact of targeted pre-activity interventions on shoulder performance in male athletes diagnosed with SIS, the study employed three distinct Intervention protocols: (1) SS, (2) DW, and (3) Combined SS and DW. All participants underwent each protocol in randomized order by one of researchers using a computerized random number generator, with a minimum 72-hour washout period between sessions to minimize carryover effects [26]. The SS protocol was designed to improve shoulder flexibility and involved six specific active stretches targeting both the dominant and non-dominant shoulder girdle musculature. Each stretch was performed in three 30-second bouts with 15 s of rest between repetitions, following the threshold-of-discomfort principle while avoiding pain [26]. Stretching movements focused on key regions such as the posterior capsule, pectoralis major, rhomboids, supraspinatus, and triceps, with form and position standardized according to prior validated protocols. The DW protocol included six upper-extremity mobility and activation exercises executed using a light-resistance yellow TheraBand^®^ (resistance level: 0.45–2.7 kg). Exercises were conducted continuously with controlled tempo (2 s per repetition), emphasizing scapular and glenohumeral mobility, neuromuscular readiness, and dynamic stabilization. Each movement was performed for 10 repetitions, with tension maintained throughout the entire ROM [26]. The combined protocol integrated three exercises from the SS set and three from the DW, executed with identical durations, sets, and sequence structures. This approach aimed synthesis the mechanical and neural benefits of both interventions. All interventions were administered under standardized conditions and supervised by assessors. A comprehensive description of each protocol is available in the Supplementary File 2.

### Outcome measures

#### Shoulder internal and external ROM assessment

Passive shoulder IR and ER ROM were assessed using a 12-inch, 360-degree goniometer. Participants were positioned in a supine lying posture with their shoulders abducted to 90 degrees and their elbows flexed at 90 degrees. The scapula was stabilized to prevent compensatory movement. For IR, the examiner passively moved the forearm anteriorly (forward) toward the floor until resistance was felt. The same goniometric alignment was used. For ER, the examiner passively moved the participant’s forearm posteriorly (backwards) in the transverse plane until the point of resistance, while ensuring the upper arm remained perpendicular to the trunk. The stationary arm of the goniometer was aligned vertically (perpendicular to the floor), and the movable arm followed the ulna, pointing toward the ulnar styloid process. All measurements were recorded in degrees [27], and each test was repeated three times per condition, with the average taken for analysis. The intra-rater reliability for this test has been previously reported as 0.94 to 0.96 for IR and 0.97 to 0.99 for ER [28].

#### Stability

The YBT for the upper quarter was used to assess shoulder stability. The test apparatus includes rods marked with measurement units in three directions and a movable indicator that is guided by the participant’s free hand. To perform the test, participants assumed a quadruped position (on their hands and toes) without wearing shoes, maintaining the alignment of their spine and lower limbs. The supporting arm was positioned such that the thumb was placed on a straight reference line, with the feet spaced shoulder-width apart. In this position, participants reached with their free hand in three specific directions: medial, superolateral, and inferolateral. They extended their reach as far as possible while maintaining the position of the supporting arm and avoiding any contact between the free hand and the ground. Reach distances were normalized to upper limb length, which was measured from the seventh cervical vertebra to the tip of the longest finger with the shoulder abducted to 90 degrees and the elbow, wrist, and fingers fully extended [29]. The test was performed sequentially in all three directions without rest. After completing all three reaches, participants were allowed to rest by placing their free hand on the ground. The procedure was repeated for three complete trials, and each participant was given one familiarization trial prior to testing [30]. The maximum reach distance in each direction was recorded and used to calculate the composite score using the following formula:

$$\begin{aligned} \mathrm{Composite}\;\mathrm{Score}\;=\;&\lbrack(\mathrm{Medial}\;\mathrm{Reach}\;+\;\mathrm{Superolateral}\;\mathrm{Reach}\;\\&+\;\mathrm{Inferolateral}\;\mathrm{Reach})\;\div\;3\rbrack\;\\&\times\;\mathrm{Upper}\;\mathrm{Limb}\;\mathrm{Length} \end{aligned}$$

The intra-rater reliability has been previously reported as 0.74 to 0.89 for this test [31].

#### JPS

To measure shoulder JPS, participants were positioned supine with their dominant arm stabilized at 90° shoulder abduction and 90° elbow flexion. Key landmarks (ulnar styloid process and olecranon) were marked with 12 mm skin-safe markers. While blindfolded, the examiner passively moved the participant’s arm to 45° IR for 5 s before returning to neutral. Participants then actively reproduced this angle. A Canon EOS 90D CMOS (1080 resolution, 60fps) positioned 3 m perpendicular to the sagittal plane captured end-range positions, with a 30 cm reference scale for calibration. Images were analyzed in AutoCAD 2023 by an assessor using vector analysis between the ulnar markers and vertical axis, with absolute error calculated as the mean difference between target and reproduced angles across three trials. The trial was repeated three times, and the mean value was used as the final record. The intra-rater reliability of this method was reported as 0.92 [32].

#### Isokinetic strength

Isokinetic shoulder strength was assessed using a Biodex dynamometer (Biodex Medical Systems, Shirley, NY, USA) at angular velocities of 60°/s and 120°/s for both IR and ER in concentric (CON) and eccentric (ECC) modes. Participants were seated upright in the dynamometer chair, with the trunk, pelvis, and tested limb stabilized using adjustable straps to minimize compensatory body movements. The shoulder was positioned in 90° abduction, with the elbow flexed to 90°, and the forearm in a neutral position. The rotational axis of the dynamometer was carefully aligned with the glenohumeral joint (humeral head) to ensure optimal biomechanical positioning and accuracy. A 30-second rest interval was provided between sets to reduce fatigue effects. The dominant limb was tested in all cases. A standardized verbal encouragement to develop maximal strength in all muscle activities was given by the principal investigator in a consistent manner to all participants during the testing procedure. Peak torque (Nm) was recorded as the primary outcome measure for each test condition [33, 34]. The average of three consistent trials was used for analysis. This procedure for assessing isokinetic shoulder rotational strength has demonstrated good to excellent intra-rater reliability in prior literature, with values ranging from 0.72 to 0.94 for both IR and ER movements [35].

### Statistical analysis

All statistical analyses were performed using SPSS software (version 27.0, IBM Corp., Armonk, NY, USA). Descriptive statistics, including means and standard deviations, were calculated for demographic and baseline characteristics. A mixed-design ANOVA was employed to examine the effects of time (baseline, post-intervention, follow-up), group (healthy, impingement), and protocol (SS, DW, SS + DW) on the dependent variables: shoulder ROM, YBT performance, JPS accuracy, and isokinetic strength measures. For each variable, we evaluated:

- Main effects of time, group, and protocol.
- Two-way interactions (time × group, protocol × group, protocol × time).
- Three-way interaction (protocol × group × time).

The assumption of sphericity was assessed using Mauchly’s test. Where violations occurred, the degrees of freedom were corrected using the Greenhouse-Geisser procedure. For any significant main effects or interactions, post-hoc testing was conducted with the Least Significant Difference test to identify specific differences. A significance level of *p* < 0.05 was used for all tests.

The effect sizes for all parameters were assessed using partial η^2^. In this context, small, medium, and large effect sizes were represented by partial η2 values of 0.02, 0.13, and 0.26, respectively [36]. Within-group Cohen’s *d* effect sizes were classified as small (0.00 < *d* < 0.49), moderate (0.50 ≤ *d* < 0.80), and large (*d* >0.80) [37]. A significance level of *p* < 0.05 was used for all tests.

## Results

### Participant characteristics

**Fig. 2:**
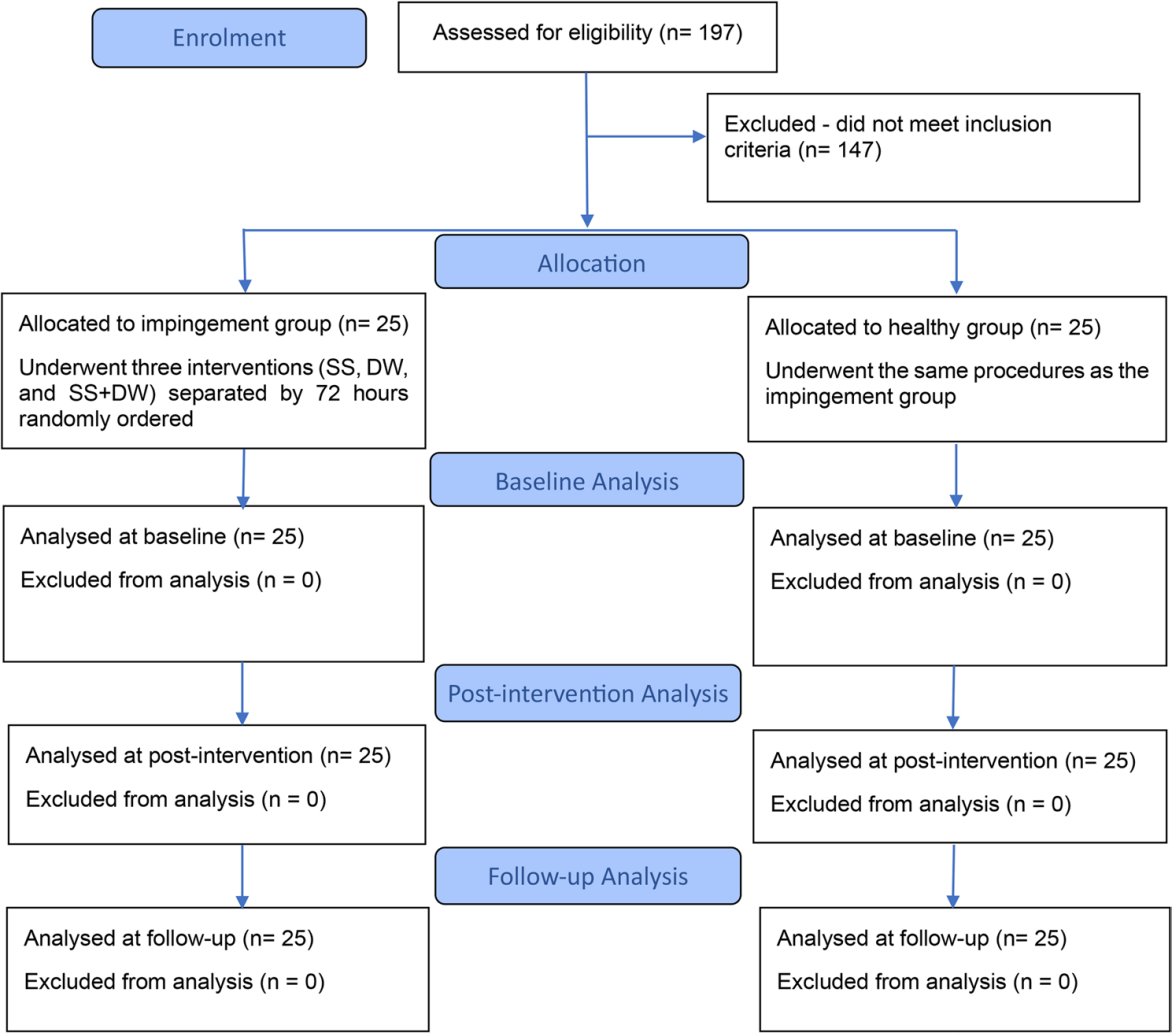
CONSORT 2025 Flow Diagram of participant enrollment, allocation, and analysis

A total of 197 athletes were initially screened for eligibility, of whom 50 participants (25 healthy controls and 25 with SIS) met the inclusion criteria and were enrolled in the study (Fig. 2). The healthy group had a mean age of 26.1 ± 1.6 years, while the impingement group had a mean age of 25.3 ± 1.4 years. Moreover, no adverse events occurred in either of the studied groups. Additional demographic characteristics are presented in Table 1. At baseline, the healthy group demonstrated significantly greater shoulder IR (*p* < 0.001) and ER (*p* < 0.001), as well as more accurate JPS (*p* < 0.001), compared to the impingement group. However, there was no significant difference in YBT performance between the two groups (*p* = 0.05). Additionally, the healthy group exhibited significantly greater isokinetic shoulder strength at both 60°/s and 120°/s (*p* < 0.001). These values are also presented in Table 1.

**Table 1:** Participants’ characteristics and baseline ROM

Variable		Healthy group (Mean ± SD)		Impingement group (Mean ± SD)		*p*-value
Age (years)		26.1 ± 1.6		25.3 ± 1.4		0.08
Height (cm)		198.2 ± 3.1		196.8 ± 4.8		0.22
Mass (kg)		93.1 ± 4.0		91.4 ± 2.8		0.10
Body mass index (kg/m ^2^)		23.7 ± 1.2		23.6 ± 0.99		0.81
Sports (N)		Volleyball: 12 Handball: 8 Basketball:5		Volleyball: 13 Handball: 4 Basketball:8		
Dominant hand (N)		22 R/3 L		24 R/1 L		
Baseline outcomes						
IR (º)		54.0 ± 3.2		49.3 ± 3.4		< 0.001
ER (º)		120.6 ± 2.7		116.9 ± 3.5		< 0.001
YBT (cm)		100.0 ± 3.9		96.7 ± 7.4		0.05
JPS (º)		6.7 ± 0.4		7.3 ± 0.5		< 0.001
Isokinetic strength (60°/s)						
CON-IR (Nm)	47.2 ± 4.6		41.6 ± 5.1		< 0.001	
ECC-IR (Nm)	63.7 ± 4.3		56.6 ± 4.3		< 0.001	
CON-ER (Nm)	40.8 ± 3.0		28.1 ± 4.2		< 0.001	
ECC-ER (Nm)	38.3 ± 4.5		27.7 ± 3.3		< 0.001	
Isokinetic strength (120°/s)						
CON-IR (Nm)	42.5 ± 3.4		35.6 ± 5.0		< 0.001	
ECC-IR (Nm)	60.6 ± 4.7		51.5 ± 3.7		< 0.001	
CON-ER (Nm)	29.8 ± 2.6		21.9 ± 4.5		< 0.001	
ECC-ER (Nm)	30.8 ± 3.1		24.4 ± 5.2		< 0.001	

*N* number, *SD* standard deviation, *R* right, *L* left, *IR* internal rotation, *ER* external rotation, *YBT* Y balance test, *JPS* joint position sense, *CON* concentric, *ECC* eccentric

### Shoulder ROM

#### IR

For IR-ROM, there were significant main effects of time, group, and protocol (all *p* < 0.05). Across participants, IR-ROM increased from baseline to post-intervention, with the SIS group showing greater improvements (SS + 4.4°, *d* = 1.25; DW + 1.9°, *d* = 0.50; SS + DW + 1.8°, *d* = 0.62; large to moderate) than the healthy group (SS + 3.0°, *d* = 1.12; DW + 1.9°, *d* = 0.54; SS + DW + 0.9°, *d* = 0.31; large to small). Despite these gains, healthy athletes consistently maintained higher overall values (54.0° vs. 49.3° at baseline, *p* < 0.001). Interaction effects indicated that athletes with SIS benefited more from SS than healthy controls, and improvements under SS were more sustained at follow-up compared to DW. The three-way interaction (protocol × group × time) was not significant (*p* > 0.05) (Tables 2 and 3).

**Table 2:** Descriptive data for outcome measures across assessment time points

Group	Protocol	Time	IR-ROM (º)	ER-ROM (º)	YBT (cm)	JPS (º)
Healthy	SS	Baseline	53.1 ± 3.5	121.2 ± 3.2	97.8 ± 5.0	6.5 ± 0.5
		Post	56.1 ± 1.4^*,**^	123.1 ± 3.1^*,**^	98.2 ± 4.9^*,**^	7.4 ± 0.5^*,**^
		Follow-up	54.6 ± 2.1^***^	122.2 ± 3.1^***^	98.0 ± 4.8^***^	6.7 ± 0.9
	DW	Baseline	54.0 ± 3.9	120.6 ± 3.5	97.9 ± 4.2	6.9 ± 0.5
		Post	55.9 ± 3.0^*,**^	122.2 ± 3.1^*,**^	100.1 ± 4.6^*^	4.6 ± 0.2^*,**^
		Follow-up	54.9 ± 3.4^***^	121.5 ± 3.4^***^	99.0 ± 4.9	5.5 ± 0.5^***^
	SS + DW	Baseline	55.4 ± 2.9	121.5 ± 1.6	97.6 ± 5.5	6.6 ± 0.5
		Post	56.3 ± 2.8^*,**^	121.9 ± 1.4^*,**^	99.2 ± 3.2	5.2 ± 0.5^*,**^
		Follow-up	56.0 ± 2.8^***^	121.7 ± 1.5^***^	98.2 ± 5.4	6.4 ± 0.5
Impingement	SS	Baseline	48.8 ± 4.1	115.7 ± 3.4	94.9 ± 7.2	7.2 ± 0.3
		Post	53.2 ± 2.8^*,**^	119.1 ± 3.2^*,**^	95.5 ± 7.0^*^	8.4 ± 0.5^*,**^
		Follow-up	50.0 ± 3.8^***^	116.8 ± 3.2^***^	95.4 ± 6.8^***^	7.4 ± 0.5
	DW	Baseline	48.7 ± 3.7	116.6 ± 3.0	94.5 ± 8.6	7.2 ± 0.4
		Post	50.6 ± 3.8^*,**^	118.2 ± 2.9^*,**^	101.9 ± 4.1^*,**^	3.4 ± 0.7^*,**^
		Follow-up	49.4 ± 3.8^***^	117.5 ± 3.0^***^	97.9 ± 4.9^***^	5.3 ± 0.7^***^
	SS + DW	Baseline	49.0 ± 3.0	116.0 ± 4.8	94.2 ± 7.0	7.6 ± 0.6
		Post	50.8 ± 2.8^*,**^	116.4 ± 4.3	95.8 ± 4.5	5.6 ± 0.7^*,**^
		Follow-up	49.6 ± 2.7^***^	161.1 ± 4.6	95.6 ± 4.1	6.9 ± 0.5^***^

Values are presented as Mean ± SD. SS, static stretching; DW, dynamic warm-up; SS + DW, combination of static stretching and dynamic warm-up; IR, internal rotation; ROM, range of motion; ER, external rotation; YBT, Y-balance test; JPS, joint position test

* means significant difference between post-intervention and baseline (p < 0.05); ** means significant difference between post-intervention and follow-up (p < 0.05); *** means significant difference between follow-up and baseline (p < 0.05)

**Table 3:** Mixed ANOVA results for measured variables

Effect	IR-ROM	ER-ROM	YBT	JPS
Time	F = 5.76 *p* = 0.006 η^2^ = 0.20	F = 3.37 *p* = 0.04 η^2^ = 0.12	F = 15.07 *p* = < 0.001 η^2^ = 0.39	F = 2.77 *p* = 0.07 η^2^ = 0.10
Group	F = 39.33 *p* = < 0.001 η^2^ = 0.45	F = 35.63 *p* = < 0.001 η^2^ = 0.43	F = 9.27 *p* = 0.004 η^2^ = 0.16	F = 28.08 *p* = < 0.001 η^2^ = 0.37
Protocol	F = 6.24 *p* = 0.004 η^2^ = 0.21	F = 1.61 *p* = 0.20 η^2^ = 0.06	F = 2.00 *p* = 0.14 η^2^ = 0.08	F = 12.81 *p* = < 0.001 η^2^ = 0.35
Group × Time	F = 4.91 *p* = 0.01 η^2^ = 0.17	F = 6.86 *p* = 0.002 η^2^ = 0.23	F = 2.21 *p* = 0.12 η^2^ = 0.08	F = 10.75 *p* = < 0.001 η^2^ = 0.31
Protocol × Time	F = 3.28 *p* = 0.01 η^2^ = 0.23	F = 1.43 *p* = 0.24 η^2^ = 0.11	F = 6.89 *p* = < 0.001 η^2^ = 0.38	F = 11.53 *p* = < 0.001 η^2^ = 0.51
Protocol × Group	F = 7.18 *p* = 0.002 η^2^ = 0.23	F = 0.02 *p* = 0.97 η^2^ = 0.001	F = 1.47 *p* = 0.24 η^2^ = 0.06	F = 61.98 *p* = < 0.001 η^2^ = 0.72
Protocol × Group × Time	F = 1.52 *p* = 0.21 η^2^ = 0.12	F = 5.92 *p* = < 0.001 η^2^ = 0.35	F = 3.60 *p* = 0.01 η^2^ = 0.24	F = 14.72 *p* = < 0.001 η^2^ = 0.57

*IR* internal rotation, *ROM* range of motion, *ER* external rotation, *YBT* Y-balance test, *JPS* joint position test

#### ER

For ER- ROM, there were significant main effects of time and group (both *p* < 0.05), while the main effect of protocol was not significant (*p* > 0.05). Within-group analyses revealed that both groups showed improvement from baseline to post-intervention. The SS protocol produced the largest gains, with the SIS group showing a substantial increase (+ 3.4°, *d* = 1.02), while the healthy group also improved (+ 1.9°, *d* = 0.60). The DW protocol resulted in moderate improvements for SIS (+ 1.6°, *d* = 0.54) and small improvements for healthy athletes (+ 1.6°, *d* = 0.48). Minimal changes were observed under the SS + DW condition in both groups (SIS + 0.4°, *d* = 0.08; healthy + 0.4°, *d* = 0.26). Nonetheless, healthy athletes consistently demonstrated higher values (120.6° vs. 116.9° at baseline, *p* < 0.001). Significant time × group and protocol × group × time interactions (*p* < 0.05) indicated that improvements were more pronounced in the SIS group, particularly under SS (Tables 2 and 3).

### YBT

For YBT performance, there were significant main effects of time and group (both *p* < 0.05), but the main effect of protocol was not significant (*p* > 0.05). Within-group analyses revealed that both groups showed improvement from baseline to post-intervention. The DW protocol produced the largest gains, with the SIS group showing a increase in reach distance (+ 7.4 cm; *d* = 1.09) and the healthy group also improving substantially (+ 2.2 cm; *d* = 0.49). The SS and SS + DW protocols resulted in small improvements for the SIS group (SS + 0.6 cm, *d* = 0.08; SS + DW + 1.6 cm, *d* = 0.27) and the healthy group (SS + 0.4 cm, *d* = 0.08; SS + DW + 1.6 cm, *d* = 0.35). Overall, healthy athletes maintained superior performance (100.0 cm vs. 96.7 cm at baseline, *p* = 0.05). Significant protocol × time and protocol × group × time interactions confirmed that YBT improvements varied by intervention (*p* < 0.05), with DW yielding the most pronounced post-intervention effects (Tables 2 and 3).

### JPS

For JPS accuracy, there were significant main effects of group and protocol (both *p* < 0.05). Healthy athletes consistently demonstrated lower error values (6.7° vs. 7.3° at baseline, *p* < 0.001). Protocol effects showed that SS increased large errors in both groups (healthy + 0.9°, *d* = 1.80; SIS + 1.2°, *d* = 2.91), whereas DW improved large accuracy (healthy − 2.3°, *d* = 6.04; SIS − 3.8°, *d* = 6.66). The SS + DW protocol also produced improved large results (healthy − 1.4°, *d* = 2.79; SIS − 2°, *d* = 3.06). Significant two-way and three-way interactions indicated that athletes with SIS gained the greatest benefit from DW (Tables 2 and 3).

### Isokinetic strength at 60°/s

At 60°/s, there were significant main effects of group (*p* < 0.05), with healthy athletes producing greater strength than athletes with SIS across all measures. Within-group analyses demonstrated that strength changes were small for CON-IR (healthy: SS − 0.5 Nm, *d* = 0.09; DW − 0.3 Nm, *d* = 0.06; SS + DW + 0.3 Nm, *d* = 0.08 and SIS: SS − 0.5 Nm, *d* = 0.09; DW − 0.2 Nm, *d* = 0.04; SS + DW + 0.5 Nm, *d* = 0.10). For ECC-IR, the SS protocol reduced strength in both groups with small to moderate effect sizes (healthy − 1.9Nm, d = 0.47; SIS – 2.5 Nm, *d* = 0.69), whereas DW (healthy + 0.8 Nm, *d* = 0.20; SIS + 0.9 Nm, *d* = 0.21) and SS + DW (healthy − 0.1Nm, *d* = 0.02; SIS + 0.1 Nm, *d* = 0.02) had small changes. For CON-ER, DW produced the small gains in the SIS group (+ 0.6 Nm, *d* = 0.13), while healthy athletes also improved (+ 0.7 Nm, *d* = 0.21) with a small effect size. Under the SS protocol, strength changes were small (SIS + 0.1 Nm, *d* = 0.02; healthy − 0.6 Nm, d = 0.15). Similarly, the SS + DW condition yielded small improvements (SIS + 0.3 Nm, *d* = 0.08; healthy + 0.5 Nm, *d* = 0.17). For ECC-ER, the SS protocol reduced strength in both groups (SIS − 0.5 Nm, d = 0.69; healthy – 1.9 Nm, *d* = 0.47) with effect sizes from small to moderate. In contrast, DW changed values in both SIS (+ 0.5 Nm, *d* = 0.15) and healthy (+ 0.2, *d* = 0.03) athletes with small effect sizes. The SS + DW condition also maintained performance, with small variations in either group (SIS + 0.2 Nm, *d* = 0.05; healthy + 0.2 Nm, *d* = 0.04). Overall, healthy athletes maintained superior strength across conditions. Significant protocol effects and protocol × group interactions (*p* < 0.05) indicated that SIS athletes were more negatively affected by SS but benefited more from DW, particularly in terms of ER strength (Tables 4 and 5).

**Table 4:** Descriptive statistics for CON and ECC shoulder rotation

Group	Protocol	Time	CON IR 60°/s	CON IR 120°/s	CON ER 60°/s	CON ER 120°/s	ECC IR 60°/s	ECC IR 120°/s	ECC ER 60°/s	ECC ER 120°/s
Healthy	SS	Baseline	47.5 ± 6.9	43.4 ± 6.8	41.2 ± 3.6	29.3 ± 5.9	62.8 ± 5.2	60.2 ± 7.1	37.6 ± 3.9	30.7 ± 3.7
		Post	47.0 ± 3.0	42.8 ± 4.2	40.6 ± 3.9^*^	27.9 ± 4.9	60.9 ± 2.3	58.3 ± 5.2	37.0 ± 3.3^*,**^	30.3 ± 2.9
		Follow-up	47.4 ± 4.6	43.1 ± 5.8	40.7 ± 3.8^***^	28.9 ± 4.8	62.0 ± 3.6	59.2 ± 3.1	37.3 ± 3.5	29.6 ± 4.9
	DW	Baseline	48.1 ± 4.8	43.3 ± 3.1	41.0 ± 3.1	29.5 ± 5.4	63.1 ± 3.6	61.3 ± 4.4	38.2 ± 5.9	30.6 ± 4.0
		Post	47.8 ± 4.6	44.5 ± 5.2	41.7 ± 3.3^*,**^	30.5 ± 2.7	63.9 ± 4.3	62.0 ± 6.0	38.4 ± 5.0	31.6 ± 4.4
		Follow-up	47.7 ± 2.7	43.9 ± 4.9	41.2 ± 3.2	29.5 ± 2.6	63.2 ± 3.9	61.6 ± 5.2	38.5 ± 5.2	30.4 ± 5.3
	SS + DW	Baseline	47.7 ± 3.5	43.6 ± 3.8	40.8 ± 2.7	29.8 ± 2.6	62.8 ± 4.7	60.2 ± 5.2	38.3 ± 4.9	30.8 ± 2.5
		Post	48.0 ± 3.6	42.8 ± 6.6	41.3 ± 3.1^*,**^	28.9 ± 3.7	62.7 ± 2.7	60.6 ± 5.0	38.5 ± 5.0^*,**^	31.3 ± 3.3
		Follow-up	47.9 ± 3.9	43.3 ± 5.9	41.1 ± 2.9^***^	29.7 ± 2.2	62.9 ± 3.6	60.5 ± 5.6	38.2 ± 4.9	30.8 ± 4.3
Impingement	SS	Baseline	41.5 ± 5.7	35.9 ± 5.5	28.6 ± 5.2	21.9 ± 4.5	56.4 ± 3.2	50.7 ± 6.1	27.9 ± 4.2	25.0 ± 6.5
		Post	41.0 ± 5.1	36.3 ± 6.9	28.7 ± 4.4	21.2 ± 5.2	53.9 ± 4.0^*^	49.5 ± 4.1	27.6 ± 3.8	25.1 ± 3.7
		Follow-up	41.4 ± 3.3	36.0 ± 4.6	28.8 ± 4.7	22.3 ± 4.3	56.2 ± 5.5	50.5 ± 4.0	27.7 ± 4.0^***^	25.2 ± 4.1
	DW	Baseline	42.0 ± 4.5	35.7 ± 5.6	28.5 ± 4.5	21.8 ± 3.1	56.6 ± 4.9	51.2 ± 3.4	27.1 ± 3.6	25.7 ± 4.5
		Post	41.8 ± 4.6	36.4 ± 4.2	29.1 ± 4.4^*,**^	22.7 ± 2.2	57.5 ± 3.5	51.5 ± 4.8	27.6 ± 2.7	25.8 ± 5.5
		Follow-up	41.7 ± 5.2	36.1 ± 5.0	28.7 ± 4.5	22.2 ± 3.7	56.9 ± 4.9	51.8 ± 4.7	27.4 ± 3.1	25.9 ± 4.3
	SS + DW	Baseline	41.4 ± 4.8	35.5 ± 5.1	27.8 ± 3.4	21.5 ± 5.3	56.9 ± 4.6	51.0 ± 3.7	27.6 ± 3.8	25.6 ± 4.5
		Post	41.9 ± 4.5	36.2 ± 4.6	28.1 ± 3.4^*^	22.1 ± 3.8	57.0 ± 5.0	51.8 ± 5.3	27.8 ± 3.7^**^	25.9 ± 3.5
		Follow-up	41.7 ± 3.0	35.7 ± 7.5	28.0 ± 3.3	21.3 ± 2.9	57.0 ± 2.6	51.6 ± 3.9	27.5 ± 3.5	25.5 ± 3.8

Values are presented as Mean ± SD. SS, static stretching; DW, dynamic warm-up; SS + DW, combination of static stretching and dynamic warm-up; CON, concentric; ECC, eccentric; IR, internal rotation; ER, external rotation

* means significant difference between post-intervention and baseline (p < 0.05); ** means significant difference between post-intervention and follow-up (p < 0.05); *** means significant difference between follow-up and baseline (p < 0.05)

**Table 5:** Mixed ANOVA results for isokinetic strength measures across speed, muscle activity’s types, and rotations

Muscle activity’s type	Rotation	Speed (º/s)	Time	Group	Protocol	Group × Time	Protocol × Time	Protocol × Group	Protocol × Group × Time
CON	IR	60	F = 1.41 *p* = 0.25 η^2^ = 0.05	F = 137.51 *p* = < 0.001 η^2^ = 0.74	F = 3.03 *p* = 0.05 η^2^ = 0.11	F = 0.15 *p* = 0.85 η^2^ = 0.007	F = 3.53 *p* = 0.01 η^2^ = 0.24	F = 0.52 *p* = 0.59 η^2^ = 0.02	F = 0.54 *p* = 0.70 η^2^ = 0.04
CON	ER	60	F = 1.17 *p* = 0.31 η^2^ = 0.04	F = 36.05 *p* = < 0.001 η^2^ = 0.43	F = 1.03 *p* = 0.36 η^2^ = 0.04	F = 0.63 *p* = 0.53 η^2^ = 0.02	F = 4.44 *p* = 0.004 η^2^ = 0.28	F = 0.32 *p* = 0.72 η^2^ = 0.01	F = 2.40 *p* = 0.06 η^2^ = 0.18
ECC	IR	60	F = 0.15 *p* = 0.85 η^2^ = 0.007	F = 193.01 *p* = < 0.001 η^2^ = 0.80	F = 4.27 *p* = 0.02 η^2^ = 0.15	F = 0.12 *p* = 0.88 η^2^ = 0.006	F = 0.90 *p* = 0.35 η^2^ = 0.09	F = 0.97 *p* = 0.59 η^2^ = 0.02	F = 0.95 *p* = 0.67 η^2^ = 0.05
ECC	ER	60	F = 0.81 *p* = 0.44 η^2^ = 0.03	F = 153.09 *p* = < 0.001 η^2^ = 0.76	F = 12.34 *p* = < 0.001 η^2^ = 0.34	F = 0.56 *p* = 0.57 η^2^ = 0.02	F = 1.98 *p* = 0.11 η^2^ = 0.15	F = 4.76 *p* = 0.01 η^2^ = 0.17	F = 0.41 *p* = 0.79 η^2^ = 0.03
CON	IR	120	F = 0.09 *p* = 0.90 η^2^ = 0.004	F = 220.21 *p* = < 0.001 η^2^ = 0.82	F = 0.42 *p* = 0.65 η^2^ = 0.01	F = 0.14 *p* = 0.86 η^2^ = 0.006	F = 0.22 *p* = 0.92 η^2^ = 0.02	F = 0.26 *p* = 0.76 η^2^ = 0.01	F = 0.15 *p* = 0.96 η^2^ = 0.01
CON	ER	120	F = 0.03 *p* = 0.96 η^2^ = 0.002	F = 195.70 *p* = < 0.001 η^2^ = 0.80	F = 1.59 *p* = 0.21 η^2^ = 0.06	F = 0.42 *p* = 0.65 η^2^ = 0.01	F = 1.05 *p* = 0.38 η^2^ = 0.08	F = 0.39 *p* = 0.67 η^2^ = 0.01	F = 0.39 *p* = 0.81 η^2^ = 0.03
ECC	IR	120	F = 0.15 *p* = 0.85 η^2^ = 0.007	F = 453.52 *p* = < 0.001 η^2^ = 0.90	F = 4.53 *p* = 0.01 η^2^ = 0.16	F = 0.07 *p* = 0.92 η^2^ = 0.003	F = 0.87 *p* = 0.47 η^2^ = 0.07	F = 0.54 *p* = 0.58 η^2^ = 0.02	F = 0.05 *p* = 0.99 η^2^ = 0.005
ECC	ER	120	F = 0.39 *p* = 0.67 η^2^ = 0.01	F = 98.18 *p* = < 0.001 η^2^ = 0.67	F = 0.98 *p* = 0.38 η^2^ = 0.04	F = 0.28 *p* = 0.75 η^2^ = 0.01	F = 0.08 *p* = 0.98 η^2^ = 0.007	F = 0.04 *p* = 0.95 η^2^ = 0.002	F = 0.13 *p* = 0.97 η^2^ = 0.01

*CON* concentric, *ECC* eccentric, *IR* internal rotation, *ER* external rotation

### Isokinetic strength at 120°/s

At 120°/s, there were significant main effects of group (*p* < 0.05), with healthy athletes consistently producing greater strength than SIS athletes across all measures. For CON-IR, changes were small in both groups (healthy: SS − 0.6 Nm, *d* = 0.10; DW + 1.2 Nm, *d* = 0.28; SS + DW − 0.8 Nm, *d* = 0.14; SIS: SS + 0.4 Nm, *d* = 0.06; DW + 0.7 Nm, *d* = 0.14; SS + DW + 0.7 Nm, *d* = 0.14). For ECC-IR, the SS protocol reduced strength with small effect sizes (healthy − 1.9 Nm, *d* = 0.30; SIS − 1.2 Nm, *d* = 0.23), while DW improved values with small effect sizes (healthy + 0.7 Nm, *d* = 0.13; SIS + 0.3 Nm, *d* = 0.07). SS + DW also changed performance small (healthy + 0.4 Nm, *d* = 0.07; SIS + 0.8 Nm, *d* = 0.17). For CON-ER, both groups showed small gains. Healthy athletes changed the strength (SS − 1.4 Nm, *d* = 0.25; DW + 1.0 Nm, *d* = 0.23; SS + DW − 0.9 Nm, *d* = 0.28), while SIS athletes also demonstrated small changes (SS − 0.7 Nm, *d* = 0.14; DW + 0.9 Nm, *d* = 0.33; SS + DW + 0.6 Nm, *d* = 0.13). For ECC-ER, strength changed small under SS (healthy − 0.4 Nm, *d* = 0.12; SIS + 0.1 Nm, *d* = 0.01), whereas DW improved small values (healthy + 1.0 Nm, *d* = 0.23; SIS + 0.1 Nm, d = 0.01). SS + DW similarly improved performance with small changes (healthy + 0.5 Nm, *d* = 0.17; SIS + 0.3 Nm, d = 0.07). Overall, healthy athletes maintained superior performance at 120°/s across all conditions. Protocol effects confirmed that DW outperformed SS, particularly for ECC-IR, by supporting small improvements in SIS athletes, whereas SS tended to reduce strength. However, no significant time effects were detected, indicating stable group differences regardless of protocol (Tables 4 and 5).

## Discussion

This RCCT compared the acute effects of SS, DW, and a combined protocol (SS + DW) on a comprehensive set of shoulder function metrics in young adult male athletes with and without SIS. The key findings demonstrate a complex interplay between intervention type, patient population, and the specific outcome measured. In summary, all three protocols produced acute improvements in ROM, while improvements in dynamic stability were most evident following DW. The protocols had divergent effects on neuromuscular control and strength, with DW generally supporting proprioceptive accuracy and preserving strength, and SS tending to reduce them. The healthy control group consistently outperformed the SIS group at baseline, and although SIS athletes often showed greater relative improvements, significant absolute deficits remained post-intervention.

### ROM

Our findings that all three protocols (SS, DW, and SS + DW) significantly improved both IR and ER ROM align with existing literature. The observation that SS provided the greatest gains in IR-ROM (SIS + 4.4°, healthy + 3.0°) and ER-ROM (SIS + 3.4°, healthy + 1.9°) is consistent with the work of Busch et al. (2021), who reported that both SS and elastic band exercises can acutely enhance shoulder mobility [9]. The mechanism is likely mechanical, as SS induces viscoelastic stress relaxation in the musculotendinous unit, leading to increased compliance and greater passive range [7]. The fact that athletes with SIS showed larger absolute improvements is clinically pertinent; it suggests that their pre-existing ROM deficits, potentially linked to posterior capsule tightness and GIRD, represent a modifiable impairment [38]. This supports the rationale for incorporating stretching into rehabilitation programs aimed at restoring glenohumeral arthrokinematics and increasing the subacromial space, as advocated by Jimenez-Del-Barrio et al. (2022) and Pıçak & Yesılyaprak (2023) [14, 16]. However, the partial diminishment of these gains at follow-up highlights the transient nature of acute flexibility improvements and underscores the need for consistent practice.

### Dynamic stability

The YBT performance improved in both groups following all interventions, with the DW protocol producing the most substantial gains (+ 7.4 cm in SIS, + 2.2 cm in healthy). This greater improvement with DW on a closed-chain dynamic task may reflect its ability to promote more active movement preparation compared to SS. Unlike SS, which primarily affects passive tissues, DW involves sport-specific, active movements that may enhance neuromuscular activation, core stability, and scapulothoracic rhythm, which are potentially important for optimal upper quarter function during weight-bearing activities [30, 39]. The controlled, resisted movements in the DW protocol might have facilitated improved recruitment of stabilizer muscles such as the serratus anterior and lower trapezius, which are often underactive in individuals with SIS [40, 41]. These adaptations could have contributed to more efficient force transfer through the kinetic chain and a greater functional reach distance [42]. The lack of a significant main effect for protocol but the presence of significant interactions suggests that while all warm-ups help, DW provides a distinct advantage for immediately preparing the shoulder for activities requiring integrated stability and mobility.

### JPS

The effects on proprioception revealed the most notable differences between protocols. While SS increased JPS error (+ 1.2° in SIS, + 0.9° in healthy) in both groups, DW appeared to improve it (+ 3.8° in SIS, + 2.3° in healthy). This may represent an important clinical observation, given that proprioceptive deficits are commonly reported in individuals with SIS and are thought to contribute to altered scapulohumeral rhythm and reduced dynamic joint stability [10]. The potential detrimental effect of SS is consistent with the findings of Sanati et al. (2022), who observed that SS may reduce shoulder joint proprioception, possibly due to temporary changes in muscle spindle sensitivity or sensorimotor processing [22]. In contrast, DW may enhance proprioceptive acuity by promoting neuromuscular activation and could be associated with increased corticospinal excitability as suggested by previous studies [43, 44]. The dynamic, controlled movements in the DW protocol might provide richer afferent feedback, potentially supporting more accurate sensorimotor function [45]. This immediate improvement in proprioception is crucial for athletes with SIS, as it may enhance joint stability and motor control during overhead activities, potentially reducing re-injury risk.

### Isokinetic strength

The most pronounced effects were observed in ECC-IR strength. At 60°/s, the SS protocol reduced ECC-IR torque in both the SIS group (–2.5 Nm) and the healthy group (–1.9 Nm). This finding aligns with prior evidence that SS can lead to temporary reductions in force production, possibly due to changes in muscle-tendon stiffness or neural drive [6, 7]. In contrast, the DW protocol avoided these negative effects and facilitated small improvements in CON-IR and CON-ER strength at 120°/s (+ 0.7 Nm in SIS and + 1.2 Nm in healthy for CON-IR; +0.9 Nm in SIS and + 1.0 Nm in healthy for CON-ER). These results suggest that the dynamic, resisted movements of the DW protocol may have acted as a form of post-activation performance enhancement, priming the neuromuscular system and supporting greater activation during subsequent high-velocity movements [46, 47]. For other outcomes, changes were generally small. The DW protocol either slightly improved or maintained baseline strength, whereas SS typically produced minimal changes or slight reductions in strength. This pattern was most evident for ECC contractions, which require high levels of stability and force absorption, mechanisms critical for decelerating the arm during overhead sports activities. Overall, healthy athletes maintained superior strength across all conditions and velocities. The findings indicate that while SS may temporarily impair strength, particularly during tasks that demand ECC control, DW can preserve or modestly enhance performance even in athletes with SIS.

### The combined protocol and clinical implications

The combined protocol (SS + DW) generally produced intermediate results. It mitigated the negative effects of isolated SS on strength and JPS, while providing better ROM improvements than DW alone; however, it did not outperform DW in neuromuscular parameters. This suggests that combining modalities may be a viable strategy to gain flexibility benefits while attenuating the negative neural effects of SS, consistent with the recommendations of Steuri et al. [19].

For clinicians and trainers, our findings advocate for a goal-specific approach:

- *To improve ROM*: SS, DW, or combined protocols can be used effectively.
- *To enhance neuromuscular control, proprioception, and strength for immediate performance*: DW is the superior strategy and should be prioritized in the pre-activity routine for athletes with SIS.
- *To avoid acute performance inhibition*: Isolated SS should be used cautiously or avoided immediately prior to activities requiring maximal strength, power, and precise motor control.

Building on these implications, the present findings also offer practical guidance for rehabilitation and return-to-play settings. DW protocols that incorporate controlled, resisted movements with elastic bands can be effectively integrated into both early and late phases of rehabilitation. During the early phase, DW may serve as a preparatory component before therapeutic exercises to promote neuromuscular readiness, enhance joint position awareness, and support the restoration of coordinated movement patterns. In later rehabilitation stages and during return-to-play progression, DW can be implemented as part of pre-activity routines to optimize shoulder mobility and dynamic stability without causing the temporary strength inhibition sometimes associated with SS. Clinicians, physiotherapists, and strength and conditioning specialists may therefore consider brief DW sessions emphasizing scapular stabilizers and rotator cuff activation as a bridge between rehabilitation exercises and sport-specific activities. Although this study was conducted under controlled laboratory conditions, the intervention protocols closely reflected practical athletic warm-ups. The DW exercises required only elastic bands, making them easily applicable in training and rehabilitation settings. However, external factors such as environment, supervision, and athlete motivation may influence outcomes. Future research should therefore examine these interventions under sport-specific, on-field conditions to confirm their ecological validity.

### Strengths and limitations

This study has several notable strengths. Firstly, the RCCT is a significant strength, as it controls for inter-individual variability by having each participant serve as their own control, thereby increasing the statistical power to detect true effects of the interventions. Secondly, the inclusion of both a healthy control group and a group with clinically diagnosed SIS allows for a robust comparison of how these interventions affect normal versus impaired shoulder function. Thirdly, the multifaceted assessment of outcomes, which evaluates not only ROM but also strength, balance, and JPS, provides a comprehensive overview of shoulder function that extends beyond the typical measures in similar studies. Furthermore, the protocols were highly standardized and directly supervised by assessors, ensuring high internal validity and consistent application of each intervention. Finally, the use of a mixed-design ANOVA for statistical analysis was appropriate for handling the complex, repeated-measures data generated by the crossover design.

Despite these strengths, several limitations must be acknowledged. The participant cohort consisted exclusively of male athletes, which limits the generalizability of the findings to female athletes, who may exhibit different biomechanical and neuromuscular responses. While the standardized, supervised protocol ensures internal validity, it may not fully replicate the practical, often unsupervised, environment of real-world athletic preparation, potentially affecting the ecological validity of the findings. The use of a single, light-resistance band for the DW protocol was appropriate for this acute study, but the effects of different resistance levels remain unknown and could be explored in future research. Lastly, while the 72-hour washout period was deemed sufficient to minimize carryover effects based on the acute nature of the interventions, the possibility of residual learning or neuromuscular adaptation effects cannot be entirely ruled out for all measures. In addition, the absence of standardized clinical outcome measures such as the SPADI or DASH questionnaires may limit the clinical interpretation of the observed improvements. Incorporating these scales in future studies would provide a stronger clinical context and facilitate comparison with other rehabilitation research. These limitations highlight the need for further research involving both sexes, longer-term interventions, and a broader range of athletic populations.

## Conclusion

In young adult male athletes with SIS, acute SS, DW, and their combination all improve ROM and dynamic stability. However, DW emerges as the most comprehensive intervention, uniquely enhancing proprioception and strength while avoiding the performance-inhibiting effects associated with SS. These results support the integration of dynamic, neuromuscular-focused warm-up protocols into the preventive and rehabilitative routines of overhead athletes with SIS to optimize shoulder function and stability before athletic participation.

## Supplementary Information

Supplementary Material 1

Supplementary Material 2

Supplementary Material 3

## Data Availability

The data that supports the findings of this study are available in the Zenodo data repository at [10.5281/zenodo.17167186].

## Abbreviations

ANOVA: Analysis of Variance
CON: Concentric
CONSORT: Consolidated Standards of Reporting Trials
DW: Dynamic Warm-up
ECC: Eccentric
ER: External Rotation
GIRD: Glenohumeral Internal Rotation Deficit
IR: Internal Rotation
IRCT: Iranian Registry of Clinical Trials
JPS: Joint Position Sense
RCCT: Randomized Controlled Crossover Trial
ROM: Range of Motion
SIS: Shoulder Impingement Syndrome
SPSS: Statistical Package for the Social Sciences
SS: Static Stretching
YBT: Y-Balance Test
SPADI: Shoulder Pain and Disability Index
DASH: Disabilities of the Arm, Shoulder and Hand
