# Supplementary material for: Effects of acute static stretching and dynamic warm-up protocols on shoulder function in young adult male athletes with shoulder impingement syndrome: a randomized controlled crossover trial
Source: BMC Musculoskelet Disord. 2025 Dec 20;26:1112. doi: 10.1186/s12891-025-09379-0 (PMC12751158; doi:10.1186/s12891-025-09379-0)
Supplement: Supplementary file 1 — Supplementary Material 1 [file 12891_2025_9379_MOESM1_ESM.docx]

|  | Item No | Recommendation | Page No |
| --- | --- | --- | --- |
| **Title and abstract** | 1 | (*a*) Identification as a randomised crossover trial in the title | 1 |
|  |  | (*b*) Specify a crossover design | 1 |
| Introduction | | | |
| Background/rationale | 2a | Scientific background and explanation of rationale | 3, 4 |
| Objectives | 2b | Specific objectives or hypotheses | 3, 4 |
| Methods | | | |
| Trial design | 3a | Rationale for a crossover design. Description of the design features including allocation ratio, especially the number and duration of periods, duration of washout period, and consideration of carry over effect | 4 |
| Change from protocol | 3b | Important changes to methods after trial commencement (such as eligibility criteria), with reasons | NA |
| Participants | 4a | Eligibility criteria for participants | 4 |
| Settings and location | 4b | Settings and locations where the data were collected | 4 |
| Interventions | 5 | The interventions with sufficient details to allow replication, including how and when they were actually administered | 5 and Supplementary File 2 |
| Outcomes | 6a | Completely defined prespecified primary and secondary outcome measures, including how and when they were assessed | 5, 6 |
| Changes to outcomes | 6b | Any changes to trial outcomes after the trial commenced, with reasons | NA |
| Sample size | 7a | How sample size was determined, accounting for within participant variability | 4, 5 |
| Interim analyses and stopping guidelines | 7b | When applicable, explanation of any interim analyses and stopping guidelines | NA |
| **Randomisation** | | | |
| Sequence generation | 8a | Method used to generate the random allocation sequence | 4 |
|  | 8b | Type of randomisation; details of any restriction (such as blocking and block size) | 4 |
| Allocation concealment | 9 | Mechanism used to implement the random allocation sequence§ (such as sequentially numbered containers), describing any steps taken to conceal the sequence until interventions were assigned | 4 |
| Implementation | 10 | Who generated the random allocation sequence,§ who enrolled participants, and who assigned participants to the sequence of interventions | 4 |
| Blinding | 11a | If done, who was blinded after assignment to interventions (for example, participants, care providers, those assessing outcomes) and how | 4 |
| Similarity of interventions | 11b | If relevant, description of the similarity of interventions | NA |
| Statistical methods | 12 | (*a*) Statistical methods used to compare groups for primary and secondary outcomes which are appropriate for crossover design (that is, based on within participant comparison) | 6, 7 |
|  |  | (*b*) Methods for additional analyses, such as subgroup analyses and adjusted analyses | 6 |
| Results | | | |
| Participants | 13* | (a) The numbers of participants who were randomly assigned, received intended treatment, and were analysed for the primary outcome, separately for each sequence and period | 7and Figure 2 |
|  |  | (b) No of participants excluded at each stage, with reasons, separately for each sequence and period | Figure 2 |
| Recruitment | 14* | (a) Dates defining the periods of recruitment and follow-up | 4 |
|  |  | (b) Why the trial ended or was stopped | NA |
| Baseline data | 15* | A table showing baseline demographic and clinical characteristics by sequence and period | Table 1 |
| Numbers analysed | 16 | Number of participants (denominator) included in each analysis and whether the analysis was by original assigned groups | Figure 2 |
| Outcomes and estimation | 17 | (*a*) For each primary and secondary outcome, results including estimated effect size and its precision (such as 95% confidence interval) should be based on within participant comparisons.¶ In addition, results for each intervention in each period are recommended | 7, 8 |
|  |  | (*b*) For binary outcomes, presentation of both absolute and relative effect sizes is recommended | NA |
| Ancillary analyses | 18 | Results of any other analyses performed, including subgroup analyses and adjusted analyses, distinguishing prespecified from exploratory | Supplementary File 3 |
| Harms | 19 | Describe all important harms or untended effects in a way that accounts for the design (for specific guidance, see CONSORT for harms32) | NA |
| Discussion | | | |
| Limitations | 19 | Trial limitations, addressing sources of potential bias, imprecision, and if relevant, multiplicity of analyses. Consider potential carry over effects | 10, 11 |
| Generalisability | 21 | Generalisability (external validity, applicability) of the trial findings | 10,11 |
| Interpretation | 22 | Interpretation consistent with results, balancing benefits and harms, and considering other relevant evidence | 9-11 |
| Other information | | | |
| Registration | 23 | Registration number and name of trial registry | 2 |
| Protocol | 24 | Where the full trial protocol can be accessed, if available | 2 |
| Funding | 25 | Give the source of funding and the role of the funders for the present study and, if applicable, for the original study on which the present article is based | 12 |

*Give information separately for exposed and unexposed groups.

**Note:** An Explanation and Elaboration article discusses each checklist item and gives methodological background and published examples of transparent reporting. The STROBE checklist is best used in conjunction with this article (freely available on the Web sites of PLoS Medicine at http://www.plosmedicine.org/, Annals of Internal Medicine at http://www.annals.org/, and Epidemiology at http://www.epidem.com/). Information on the STROBE Initiative is available at www.strobe-statement.org.
