# Supplementary material for: Effects of acute static stretching and dynamic warm-up protocols on shoulder function in young adult male athletes with shoulder impingement syndrome: a randomized controlled crossover trial
Source: BMC Musculoskelet Disord. 2025 Dec 20;26:1112. doi: 10.1186/s12891-025-09379-0 (PMC12751158; doi:10.1186/s12891-025-09379-0)
Supplement: Supplementary file 2 — Supplementary Material 2 [file 12891_2025_9379_MOESM2_ESM.docx]

**Supplementary Table 1.** Pairwise comparisons for IR-ROM.

| **Comparison** | **MD** | ***p*-value** | **95% CI** |
| --- | --- | --- | --- |
| **Group Effect** | | | |
| Healthy vs. Impingement | 4.71 | < 0.001 | 3.20 to 6.22 |
| **Protocol Effect** | | | |
| SS vs. DW | 0.38 | 0.35 | - 0.44 to 1.20 |
| SS vs. SS + DW | - 0.21 | 0.68 | - 1.25 to 0.82 |
| DW vs. SS + DW | - 0.59 | 0.28 | - 1.70 to 0.51 |
| **Time Effect** | | | |
| Baseline vs. Post | - 2.30 | < 0.001 | - 2.68 to - 1.92 |
| Baseline vs. Follow-up | - 0.90 | < 0.001 | - 1.09 to - 0.70 |
| Post vs. Follow-up | 1.40 | < 0.001 | 1.17 to 1.63 |

**Note:** IR, internal rotation; ROM, range of motion; SS, static stretching; DW, dynamic warm-up; SS + DW, combination of static stretching and dynamic warm-up; MD, mean difference; CI, confidence interval.

**Supplementary Table 2.** Pairwise comparisons for ER-ROM.

| **Comparison** | **MD** | ***p*-value** | **95% CI** |
| --- | --- | --- | --- |
| **Group Effect** | | | |
| Healthy vs. Impingement | 4.31 | < 0.001 | 2.86 to 5.77 |
| **Protocol Effect** | | | |
| SS vs. DW | 0.22 | 0.74 | - 1.13 to 1.58 |
| SS vs. SS + DW | 0.71 | 0.27 | - 0.57 to 2.00 |
| DW vs. SS + DW | 0.49 | 0.41 | - 0.71 to 1.69 |
| **Time Effect** | | | |
| Baseline vs. Post | - 1.55 | < 0.001 | - 1.73 to - 1.38 |
| Baseline vs. Follow-up | - 0.69 | < 0.001 | - 0.78 to - 0.60 |
| Post vs. Follow-up | 0.86 | < 0.001 | 0.73 to 0.98 |

**Note:** ER, external rotation; ROM, range of motion; SS, static stretching; DW, dynamic warm-up; SS + DW, combination of static stretching and dynamic warm-up; MD, mean difference; CI, confidence interval.

**Supplementary Table 3.** Pairwise comparisons for YBT.

| **Comparison** | **MD** | ***p*-value** | **95% CI** |
| --- | --- | --- | --- |
| **Group Effect** | | | |
| Healthy vs. Impingement | 1.92 | 0.01 | 0.36 to 3.48 |
| **Protocol Effect** | | | |
| SS vs. DW | - 1.92 | 0.06 | - 3.96 to 0.12 |
| SS vs. SS + DW | - 0.13 | 0.89 | - 2.13 to 1.87 |
| DW vs. SS + DW | 1.79 | 0.02 | 0.19 to 3.38 |
| **Time Effect** | | | |
| Baseline vs. Post | - 2.27 | < 0.001 | - 2.88 to - 1.66 |
| Baseline vs. Follow-up | - 1.19 | 0.004 | - 1.99 to - 0.40 |
| Post vs. Follow-up | 1.07 | 0.002 | 0.42 to 1.73 |

**Note:** YBT, Y balance test; SS, static stretching; DW, dynamic warm-up; SS + DW, combination of static stretching and dynamic warm-up; MD, mean difference; CI, confidence interval.

**Supplementary Table 4.** Pairwise comparisons for JPS.

| **Comparison** | **MD** | **SE** | ***p*-value** | **95% CI** |
| --- | --- | --- | --- | --- |
| **Group Effect** | | | | |
| Healthy vs. Impingement | 0.37 | 0.07 | < 0.001 | 0.23 to 0.51 |
| **Protocol Effect** | | | | |
| SS vs. DW | 1.77 | 0.05 | < 0.001 | 1.66 to 1.89 |
| SS vs. SS + DW | 0.86 | 0.07 | < 0.001 | 0.72 to 1.01 |
| DW vs. SS + DW | - 0.90 | 0.06 | < 0.001 | - 1.03 to - 0.78 |
| **Time Effect** | | | | |
| Baseline vs. Post | 1.22 | 0.06 | < 0.001 | 1.10 to 1.35 |
| Baseline vs. Follow-up | 0.64 | 0.06 | < 0.001 | 0.51 to 0.77 |
| Post vs. Follow-up | - 0.58 | 0.07 | < 0.001 | - 0.73 to - 0.43 |

**Note:** JPS, joint position sense; SS, static stretching; DW, dynamic warm-up; SS + DW, combination of static stretching and dynamic warm-up; MD, mean difference; CI, confidence interval.

**Supplementary Table 5.** Pairwise comparisons for CON-IR at 60°/s.

| **Comparison** | **MD** | **SE** | ***p*-value** | **95% CI** |
| --- | --- | --- | --- | --- |
| **Group Effect** | | | | |
| Healthy vs. Impingement | 5.37 | 0.45 | < 0.001 | 4.45 to 6.29 |
| **Protocol Effect** | | | | |
| SS vs. DW | - 0.57 | 0.52 | 0.28 | - 1.63 to 0.48 |
| SS vs. SS + DW | - 0.46 | 0.52 | 0.38 | - 1.50 to 0.58 |
| DW vs. SS + DW | 0.11 | 0.43 | 0.79 | - 0.76 to 0.99 |
| **Time Effect** | | | | |
| Baseline vs. Post | 0.10 | 0.52 | 0.83 | - 0.94 to 1.16 |
| Baseline vs. Follow-up | 0.07 | 0.50 | 0.88 | - 0.93 to 1.08 |
| Post vs. Follow-up | - 0.03 | 0.42 | 0.93 | - 0.89 to 0.82 |

**Note:** CON, concentric; IR, internal rotation; SS, static stretching; DW, dynamic warm-up; SS + DW, combination of static stretching and dynamic warm-up; MD, mean difference; CI, confidence interval.

**Supplementary Table 6.** Pairwise comparisons for ECC-IR at 60°/s.

| **Comparison** | **MD** | **SE** | ***p*-value** | **95% CI** |
| --- | --- | --- | --- | --- |
| **Group Effect** | | | | |
| Healthy vs. Impingement | 5.58 | 0.40 | < 0.001 | 4.77 to 6.39 |
| **Protocol Effect** | | | | |
| SS vs. DW | - 1.50 | 0.46 | 0.002 | - 2.44 to - 0.56 |
| SS vs. SS + DW | - 1.18 | 0.47 | 0.01 | - 2.14 to - 0.22 |
| DW vs. SS + DW | 0.31 | 0.44 | 0.48 | - 0.58 to 1.20 |
| **Time Effect** | | | | |
| Baseline vs. Post | 0.45 | 0.55 | 0.41 | - 0.65 to 1.56 |
| Baseline vs. Follow-up | 0.06 | 0.63 | 0.91 | - 1.20 to 1.33 |
| Post vs. Follow-up | - 0.39 | 0.50 | 0.44 | - 1.40 to 0.62 |

**Note:** ECC, eccentric; IR, internal rotation; SS, static stretching; DW, dynamic warm-up; SS + DW, combination of static stretching and dynamic warm-up; MD, mean difference; CI, confidence interval.

**Supplementary Table 7.** Pairwise comparisons for CON-ER at 60°/s.

| **Comparison** | **MD** | **SE** | ***p*-value** | **95% CI** |
| --- | --- | --- | --- | --- |
| **Group Effect** | | | | |
| Healthy vs. Impingement | 10.17 | 1.69 | < 0.001 | 6.76 to 13.57 |
| **Protocol Effect** | | | | |
| SS vs. DW | - 0.26 | 0.56 | 0.64 | - 1.41 to 0.87 |
| SS vs. SS + DW | 0.24 | 0.56 | 0.67 | - 0.89 to 1.38 |
| DW vs. SS + DW | 0.50 | 0.59 | 0.39 | - 0.68 to 1.70 |
| **Time Effect** | | | | |
| Baseline vs. Post | - 0.25 | 0.10 | 0.02 | - 0.46 to - 0.03 |
| Baseline vs. Follow-up | - 0.07 | 0.07 | 0.34 | - 0.22 to 0.07 |
| Post vs. Follow-up | 0.17 | 0.06 | 0.008 | 0.04 to 0.31 |

**Note:** CON, concentric; ER, external rotation; SS, static stretching; DW, dynamic warm-up; SS + DW, combination of static stretching and dynamic warm-up; MD, mean difference; CI, confidence interval.

**Supplementary Table 8.** Pairwise comparisons for ECC-ER at 60°/s.

| **Comparison** | **MD** | **SE** | ***p*-value** | **95% CI** |
| --- | --- | --- | --- | --- |
| **Group Effect** | | | | |
| Healthy vs. Impingement | 9.90 | 0.80 | < 0.001 | 8.29 to 11.51 |
| **Protocol Effect** | | | | |
| SS vs. DW | - 0.33 | 0.73 | 0.64 | - 1.80 to 1.12 |
| SS vs. SS + DW | - 0.51 | 0.74 | 0.49 | - 2.01 to - 0.99 |
| DW vs. SS + DW | - 0.17 | 0.75 | 0.82 | - 1.69 to 1.34 |
| **Time Effect** | | | | |
| Baseline vs. Post | - 0.04 | 0.07 | 0.54 | - 0.20 to 0.11 |
| Baseline vs. Follow-up | 0.008 | 0.06 | 0.90 | - 0.11 to 0.13 |
| Post vs. Follow-up | 0.05 | 0.04 | 0.24 | - 0.03 to 0.15 |

**Note:** ECC, eccentric; ER, external rotation; SS, static stretching; DW, dynamic warm-up; SS + DW, combination of static stretching and dynamic warm-up; MD, mean difference; CI, confidence interval.

**Supplementary Table 9**. Pairwise comparisons for CON-IR at 120°/s.

| **Comparison** | **MD** | **SE** | ***p*-value** | **95% CI** |
| --- | --- | --- | --- | --- |
| **Group Effect** | | | | |
| Healthy vs. Impingement | 7.45 | 0.50 | < 0.001 | 6.44 to 8.46 |
| **Protocol Effect** | | | | |
| SS vs. DW | - 0.42 | 0.58 | 0.47 | - 1.59 to 0.75 |
| SS vs. SS + DW | 0.05 | 0.72 | 0.94 | - 1.41 to 1.51 |
| DW vs. SS + DW | 0.47 | 0.61 | 0.44 | - 0.76 to 1.70 |
| **Time Effect** | | | | |
| Baseline vs. Post | - 0.28 | 0.63 | 0.65 | - 1.55 to 0.98 |
| Baseline vs. Follow-up | - 0.15 | 0.67 | 0.82 | - 1.51 to 1.20 |
| Post vs. Follow-up | 0.12 | 0.56 | 0.82 | - 1.00 to 1.26 |

**Note:** CON, concentric; IR, internal rotation; SS, static stretching; DW, dynamic warm-up; SS + DW, combination of static stretching and dynamic warm-up; MD, mean difference; CI, confidence interval.

**Supplementary Table 10.** Pairwise comparisons for ECC-IR at 120°/s.

| **Comparison** | **MD** | **SE** | ***p*-value** | **95% CI** |
| --- | --- | --- | --- | --- |
| **Group Effect** | | | | |
| Healthy vs. Impingement | 9.37 | 0.44 | < 0.001 | 8.49 to 10.26 |
| **Protocol Effect** | | | | |
| SS vs. DW | - 1.82 | 0.60 | 0.004 | - 3.04 to - 0.60 |
| SS vs. SS + DW | - 1.18 | 0.66 | 0.08 | - 2.51 to 0.14 |
| DW vs. SS + DW | 0.64 | 0.66 | 0.34 | - 0.69 to 1.97 |
| **Time Effect** | | | | |
| Baseline vs. Post | 0.14 | 0.52 | 0.77 | - 0.91 to 1.20 |
| Baseline vs. Follow-up | - 0.12 | 0.58 | 0.83 | - 1.30 to 1.06 |
| Post vs. Follow-up | - 0.27 | 0.49 | 0.58 | - 1.26 to 0.71 |

**Note:** ECC, eccentric; IR, internal rotation; SS, static stretching; DW, dynamic warm-up; SS + DW, combination of static stretching and dynamic warm-up; MD, mean difference; CI, confidence interval.

**Supplementary Table 11.** Pairwise comparisons for CON-ER at 120°/s.

| **Comparison** | **MD** | **SE** | ***p*-value** | **95% CI** |
| --- | --- | --- | --- | --- |
| **Group Effect** | | | | |
| Healthy vs. Impingement | 7.43 | 0.53 | < 0.001 | 6.36 to 8.50 |
| **Protocol Effect** | | | | |
| SS vs. DW | - 0.76 | 0.44 | 0.09 | - 1.66 to 0.12 |
| SS vs. SS + DW | - 0.28 | 0.53 | 0.59 | - 1.36 to 0.78 |
| DW vs. SS + DW | 0.47 | 0.46 | 0.30 | - 0.44 to 1.40 |
| **Time Effect** | | | | |
| Baseline vs. Post | 0.08 | 0.41 | 0.83 | - 0.74 to 0.92 |
| Baseline vs. Follow-up | 0.01 | 0.47 | 0.98 | - 0.95 to 0.97 |
| Post vs. Follow-up | - 0.07 | 0.36 | 0.83 | - 0.81 to 0.65 |

**Note:** CON, concentric; ER, external rotation; SS, static stretching; DW, dynamic warm-up; SS + DW, combination of static stretching and dynamic warm-up; MD, mean difference; CI, confidence interval.

**Supplementary Table 12.** Pairwise comparisons for ECC-ER at 120°/s.

| **Comparison** | **MD** | **SE** | ***p*-value** | **95% CI** |
| --- | --- | --- | --- | --- |
| **Group Effect** | | | | |
| Healthy vs. Impingement | 5.15 | 0.52 | < 0.001 | 4.10 to 6.19 |
| **Protocol Effect** | | | | |
| SS vs. DW | - 0.67 | 0.54 | 0.22 | - 1.75 to 0.41 |
| SS vs. SS + DW | - 0.64 | 0.52 | 0.22 | - 1.69 to 0.41 |
| DW vs. SS + DW | 0.02 | 0.52 | 0.95 | - 1.02 to 1.07 |
| **Time Effect** | | | | |
| Baseline vs. Post | - 0.27 | 0.47 | 0.57 | - 1.23 to 0.69 |
| Baseline vs. Follow-up | 0.17 | 0.48 | 0.72 | - 0.79 to 1.14 |
| Post vs. Follow-up | 0.44 | 0.49 | 0.38 | - 0.56 to 1.44 |

**Note:** ECC, eccentric; ER, external rotation; SS, static stretching; DW, dynamic warm-up; SS + DW, combination of static stretching and dynamic warm-up; MD, mean difference; CI, confidence interval.
