# Supplementary material for: Effects of acute static stretching and dynamic warm-up protocols on shoulder function in young adult male athletes with shoulder impingement syndrome: a randomized controlled crossover trial
Source: BMC Musculoskelet Disord. 2025 Dec 20;26:1112. doi: 10.1186/s12891-025-09379-0 (PMC12751158; doi:10.1186/s12891-025-09379-0)
Supplement: Supplementary file 3 — Supplementary Material 3 [file 12891_2025_9379_MOESM3_ESM.docx]

| **Static Stretching Protocol (SS)** | |
| --- | --- |
| **The Deltoid/Posterior Shoulder Stretch** begins with the subject standing against a wall with their dominant shoulder and the lateral border of their scapula in contact with the surface. The dominant shoulder is flexed to 80-90°, and a passive horizontal adduction force is applied by the non-dominant arm to the dominant elbow. The end position involves flexion of the dominant elbow, with the dominant hand reaching behind the opposite shoulder. By leaning against the wall, the lateral border of the scapula remains against the wall to prevent the scapula from following the humerus across the body. | 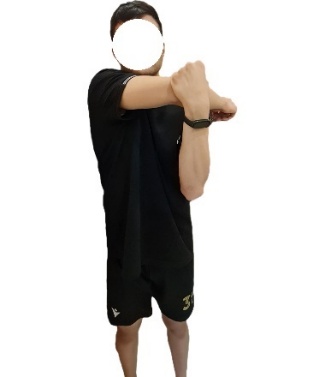 |
| **The Pec Major/Anterior Capsule Stretch** begins with the subject standing and facing a wall support. With the dominant shoulder flexed to 90° and the dominant elbow in full extension, the palm of the dominant hand makes contact against the wall. The patient then rotates their trunk away from their dominant limb while maintaining shoulder and elbow joint angles. | 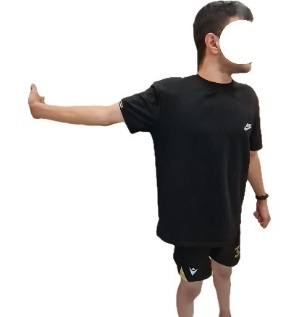 |
| **The Standing Sleeper Stretch** begins with the subject standing with their dominant shoulder against a wall support, flexed at 90°, with the elbow also in 90° of flexion. The subject then leans against the wall, applying pressure to the lateral border of their scapula. The head and neck remain in a neutral position, looking straight ahead. The scapula remains pressed against the wall while the dominant shoulder is moved into internal rotation by slowly pressing the forearm down with the non-dominant hand. | 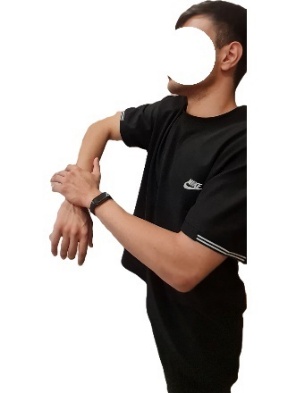 |
| **The Rhomboid/Posterior Shoulder Stretch** begins with the subject facing the edge of a door. The feet are placed on each side of the door, the hanDW on the doorknobs, and the knees in full extension. The subject then proceeDW to let the hips drop downward and backward. The elbows remain fully extended with the shoulders flexed at 90° as the body moves backward. Subjects are told to relax the arms so that the body weight stretches the rhomboid. | 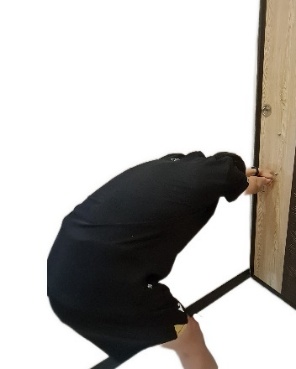 |
| **The Supraspinatus/Superior Capsule Stretch** begins with the subject standing with the dominant arm behind the body, with the elbow flexed to 90°. The subject then grasps the dominant hand with the non-dominant hand and pulls the dominant arm toward the non-dominant side. | *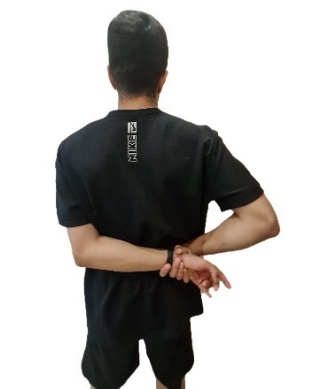* |
| **The Triceps/Inferior Capsule Stretch begins** with the subject standing with the dominant arm overhead. The subject then flexes the elbow so that the forearm is placed behind the head perpendicular to the ground. The subject then pulls downward on the elbow of the dominant arm with the hand of the non-dominant arm. | 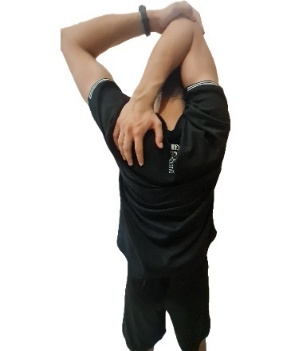 |
| **Dynamic Warm-up Protocol (DW)** | |
| **Shoulder Extension:** Standing facing towarDW the stable base with the elbow extended and the forearm in a neutral position (thumb pointing upward), the exercise begins with the subject's shoulder at 90° of forward flexion. The exercise consists of moving the shoulder toward maximum extension and then returning to the starting position while maintaining both elbow extension and the forearm-neutral position. The rubber tubing is secured to a stable base at a height equal to the height of each subject’s fingertips with the arm fully flexed in a standing position (high fixation position). | 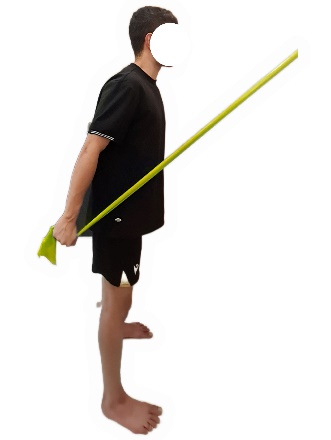 |
| **Shoulder Flexion:** Standing facing away from the stable base with the elbow extended and the forearm in a neutral position (thumb pointing upward), the exercise begins with the shoulder fully extended. The exercise consists of moving the shoulder toward maximum flexion and then returning to the starting position, while maintaining both elbow extension and the forearm-neutral position. The rubber tubing is secured to a stable base at a height equal to the height of each subject’s fingertips from the ground while standing in anatomical position (low fixation position). | 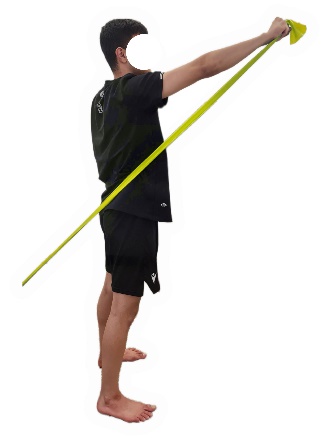 |
| **Scapular Punch:** Standing facing away from the stable base, the starting position for this exercise is with the elbow fully flexed, forearm in a neutral position, and the scapula fully retracted. The exercise consists of flexing the shoulder to approximately 100°, extending the elbow, and fully protracting the scapula while punching forward and then returning to the starting position. The rubber tubing is secured to a stable base at a height equal to the height of each subject’s elbow from the ground when standing in anatomical position (middle fixation position). | *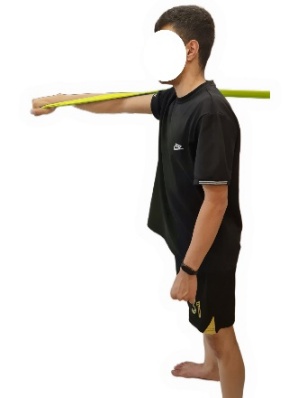* |
| **Throwing Acceleration:** Standing facing away from the stable base with the shoulder abducted and the elbow flexed to 90°, the exercise begins with the subject’s shoulder in full external rotation. The exercise consists of moving the arm across the body (similar to the acceleration phase of throwing [D2 flexion pattern]) and then returning to the starting position. The rubber tubing is secured to a stable base at a height equal to the height of each subject’s fingertips with the arm fully flexed in a standing position (high fixation position). | 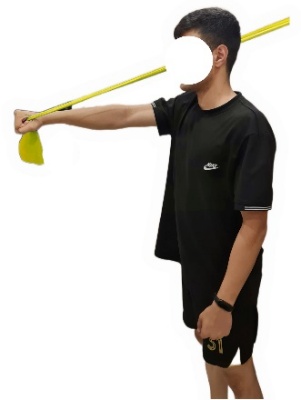 |
| **Throwing Deceleration:** When standing facing a stable base, the exercise begins with each subject's shoulder at 30° of flexion. The exercise involves pulling the tubing back so that the shoulder moves into full extension and scapular retraction. At full shoulder extension, the shoulder moves to 90° each of shoulder external rotation, shoulder abduction, and elbow flexion. The exercise finishes with the subject eccentrically controlling the tubing as the arm returns to the starting position of 30° of shoulder flexion. The rubber tubing is secured to a stable base at a height equal to the height of each subject's fingertips from the ground while standing in anatomical position (low fixation position). | 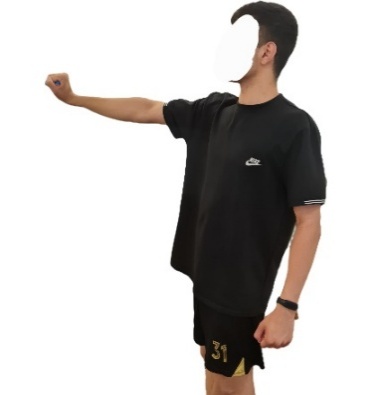 |
| **ER @ 90° of Abduction:** When standing facing a stable base with both the shoulder abducted and elbow flexed to 90°, the exercise begins with the subject's shoulder in full internal rotation. The exercise involves moving the shoulder into full external rotation and then returning to the starting position while maintaining the shoulder-abduction and elbow-flexion positions. The rubber tubing is secured to a stable base at a height equal to the height of each subject's fingertips from the ground while standing in anatomical position (low fixation position). | 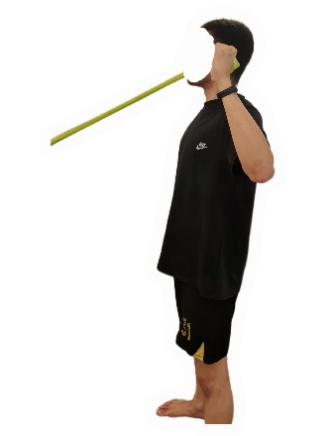 |
| **Combination of Static Stretching and Dynamic Warm-up Protocol (SS + DW)** | |
| **Shoulder Extension:** Standing facing towarDW the stable base with the elbow extended and the forearm in a neutral position (thumb pointing upward), the exercise begins with the subject's shoulder at 90° of forward flexion. The exercise consists of moving the shoulder toward maximum extension and then returning to the starting position while maintaining both elbow extension and the forearm-neutral position. The rubber tubing is secured to a stable base at a height equal to the height of each subject’s fingertips with the arm fully flexed in a standing position (high fixation position). | 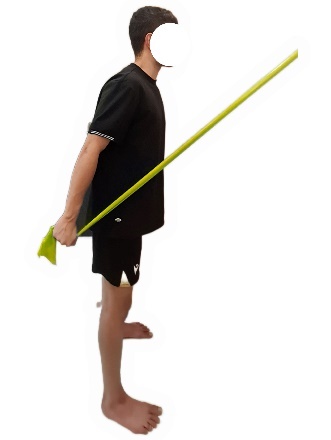 |
| **The Deltoid/Posterior Shoulder Stretch** begins with the subject standing against a wall with their dominant shoulder and the lateral border of their scapula in contact with the surface. The dominant shoulder is flexed to 80-90°, and a passive horizontal adduction force is applied by the non-dominant arm to the dominant elbow. The end position involves flexion of the dominant elbow, with the dominant hand reaching behind the opposite shoulder. By leaning against the wall, the lateral border of the scapula remains against the wall to prevent the scapula from following the humerus across the body. | 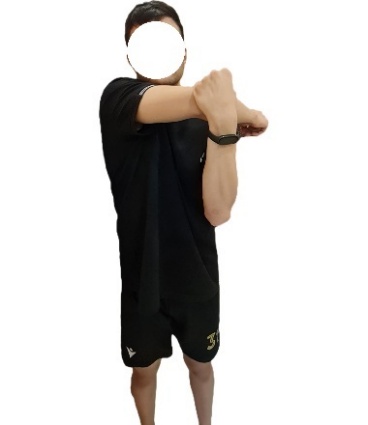 |
| **Scapular Punch:** Standing facing away from the stable base, the starting position for this exercise is with the elbow fully flexed, forearm in a neutral position, and the scapula fully retracted. The exercise consists of flexing the shoulder to approximately 100°, extending the elbow, and fully protracting the scapula while punching forward and then returning to the starting position. The rubber tubing is secured to a stable base at a height equal to the height of each subject’s elbow from the ground when standing in anatomical position (middle fixation position). | 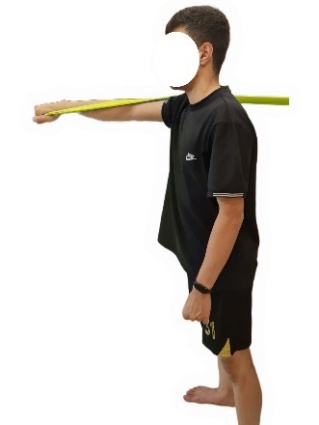 |
| **The Pec Major/Anterior Capsule Stretch** begins with the subject standing and facing a wall support. With the dominant shoulder flexed to 90° and the dominant elbow in full extension, the palm of the dominant hand makes contact against the wall. The patient then rotates their trunk away from their dominant limb while maintaining shoulder and elbow joint angles. | 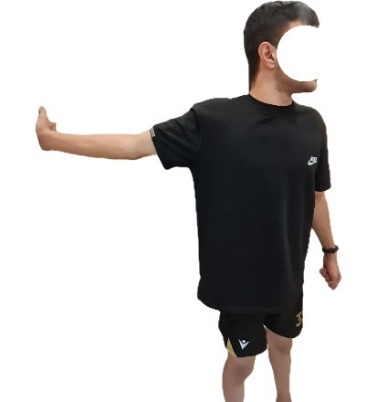 |
| **Throwing Acceleration:** Standing facing away from the stable base with the shoulder abducted and the elbow flexed to 90°, the exercise begins with the subject’s shoulder in full external rotation. The exercise consists of moving the arm across the body (similar to the acceleration phase of throwing [D2 flexion pattern]) and then returning to the starting position. The rubber tubing is secured to a stable base at a height equal to the height of each subject’s fingertips with the arm fully flexed in a standing position (high fixation position). | 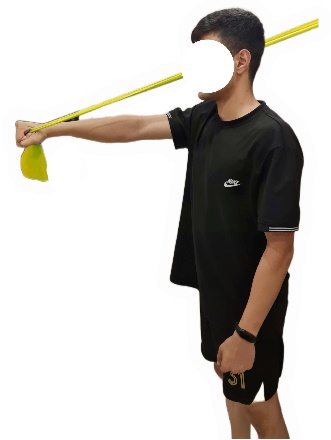 |
| **The Triceps/Inferior Capsule Stretch begins** with the subject standing with the dominant arm overhead. The subject then flexes the elbow so that the forearm is placed behind the head perpendicular to the ground. The subject then pulls downward on the elbow of the dominant arm with the hand of the non-dominant arm. | 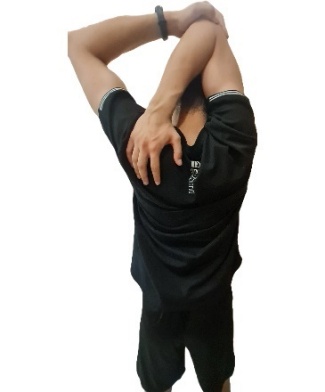 |
